# Fetid Breath

**Published:** 1897-06

**Authors:** 


### Fetid Breath.

According to a recent statement in *Sem. Med.*, ichthyol is one of the best and most rapid cures for the fetid breath of ozena. The nose is first douched with a warm 2-percent solution, and the mucous membrane is then swabbed with a 30-percent solution. This is also good in cases of rhinitis and pharyngitis sicca, without ozena.

